# Reduced inter-hemispheric auditory and memory-related network interactions in patients with schizophrenia experiencing auditory verbal hallucinations

**DOI:** 10.3389/fpsyt.2022.956895

**Published:** 2022-08-03

**Authors:** Cheng Chen, Huan Huang, Xucong Qin, Liang Zhang, Bei Rong, Gaohua Wang, Huiling Wang

**Affiliations:** ^1^Department of Psychiatry, Renmin Hospital of Wuhan University, Wuhan, China; ^2^Hubei Institute of Neurology and Psychiatry Research, Wuhan, China; ^3^Hubei Provincial Key Laboratory of Developmentally Originated Disease, Wuhan, China

**Keywords:** schizophrenia, auditory verbal hallucinations, voxel-mirrored homotopic connectivity, auditory-related network, memory-related network

## Abstract

**Background:**

Inter-hemispheric disconnection is a primary pathological finding in schizophrenia. However, given the inherent complexity of this disease and its development, it remains unclear as to whether associated inter-hemispheric changes play an important role in auditory verbal hallucination (AVH) development. As such, this study was developed to explore inter-hemispheric connectivity in the context of schizophrenia with AVH while excluding positive symptoms and other factors with the potential to confound these results.

**Method:**

In total, resting-state functional magnetic resonance imaging (fMRI) was used to assess 42 patients with AVH (APG), 26 without AVH (NPG), and 82 normal control (NC) individuals. Inter-hemispheric connectivity in these subjects was then assessed through the use of voxel-mirrored homotopic connectivity (VMHC) and Pearson correlation analyses.

**Result:**

Relative to HC and NPG subjects, APG individuals exhibited a decrease in VMHC in the superior temporal gyrus (STG) extending into Heschl's gyrus, the insula, and the Rolandic operculum as well as in the fusiform gyrus extending into the para-hippocampus (Corrected *p* < 0.005, cluster size = 52). Among APG individuals, these observed impairments of inter-hemispheric connectivity were negatively correlated with Hoffman auditory hallucination scores.

**Conclusion:**

These results support the schizophrenia hemitropic disconnection hypothesis, and provide novel evidence suggesting that there may be a relationship between reductions in inter-hemispheric connectivity in auditory and memory-related networks and the pathogenesis of AVH in patients with schizophrenia following the exclusion of confounding factors from other positive symptoms.

## Introduction

Auditory verbal hallucinations (AVHs) are a common symptom in patients diagnosed with schizophrenia, affecting up to 60–70% of these individuals (1). The pathological basis for AVH development has been linked to the hemispheric specialization of functional connectivity within the brain (2–4). As noted previously, relative increases in the contributions of right hemisphere language areas may correspond to the more complex experiential characteristics of AVHs (5), with reductions in language lateralization potentially contributing to the perceived reality of these hallucinatory sounds. Other functional magnetic resonance imaging (fMRI) studies have reported schizophrenia to be associated with decreases in the typical lateralization of language processing in the brain (left > right), with AVH severity being associated with decreased functional lateralization (6, 7). Other studies have further linked the development of schizophrenia to interhemispheric disconnectivity, with the specific localization of such abnormalities in affected patients potentially playing a role in AVH development (8–10). At present, it remains unclear as to whether left-greater-than-right or right-greater-than-left lateralization is most closely associated with AVH development. Differences in interhemispheric connectivity that have previously been observed when comparing patients with and without AVHs may also be confounded by other positive symptoms such as excitement, delusions, or confusion. Given this possibility and inconsistencies among prior studies, there is a clear need to conduct further studies exploring the relationships between changes within and between hemispheres and AVH incidence in individuals with schizophrenia. We supposed that there might be a relationship between reductions in inter-hemispheric connectivity in auditory and memory-related networks and the pathogenesis of AVH in patients with schizophrenia.

The present study was developed to assess interhemispheric connectivity in schizophrenia patients with and without AVHs through the use of a voxel-mirrored homotopic connectivity (VMHC) approach in order to establish the relationship between interhemispheric changes and AVH development. The results of these analyses will serve as the first exploration of the mechanistic basis for AVH incidence in patients with schizophrenia as a function of inter-hemispheric connectivity when analyzed in a manner that minimized the confounding effects of other positive symptoms and related clinical symptoms on associated phenotypes.

## Materials and methods

### Study participants

In total, 68 individuals diagnosed with schizophrenia were recruited from the inpatients of the Psychiatry department of Renmin Hospital of Wuhan University (Wuhan, China). Diagnoses were confirmed by administering the Structured Clinical Interview for the Diagnostic and Statistical Manual of Mental Disorder (SCID), 4th edition (DSM-IV). These patients were further separated into two groups based on whether they do or do not experience AVHs (42 APG and 26 NPG, respectively). Classification standards were based upon PANSS scores and details pertaining to current and past symptoms obtained through a combination of face-to-face interviews and a review of prior medical records. Patients were included in the APG group if they exhibited a P3 (hallucination) score of > 4 and reported experiencing AVHs a minimum of once per month. APG status was additionally assessed by Professor HL Wang using the Hoffman Auditory Hallucination scale so as to establish the content, frequency, emotional impact, severity, and degree of attention associated with these hallucinations (11). Participants in the NPG group were patients that had not experienced AVH during the course of their illness. In addition, a normal control (NC) group consisting of 82 age-, sex-, and handedness (right-handed)-matched native Chinese speakers were recruited for this study, which received approval from the local research ethics committee. All subjects provided written informed consent to participate following the review of a complete study description. Patient clinical and demographical data are compiled in Table 1.

**Table 1:** Demographics and clinical characteristics of the participants.

	**NC (*n* = 82)**	**APG (*n* = 42)**	**NPG (*n* = 26)**	***P***
Age	24.67 ± 4.71	24.64 ± 5.24	25.31 ± 5.51	0.836^a^
Gender (M/F)	82 (41/41)	42 (19/23)	42 (16/10)	0.419^b^
Education (year)	14.01 ± 1.84	12.33 ± 2.66	12.23 ± 3.29	0.000^a^
Illness duration (month)		39.24 ± 42.09	51.50 ± 60.33	0.652
Medicine		408.33 ± 220.01	397.12 ± 190.57	0.284
PANSS total		86.05 ± 12.10	84.35 ± 9.98	0.603
PANSS positive score		23.24 ± 3.56	21.70 ± 5.14	0.100
P1 (delusion)		5.02 ± 0.95	5.35 ± 1.16	0.146
P2 (conception confusion)		1.50 ± 0.74	2.15 ± 0.97	0.098
P3 (hallucination)		5.10 ± 0.96	0.92 ± 0.56	0.000^*^
P4 (excitement)		2.05 ± 1.10	2.54 ± 0.86	0.569
P5 (exaggeration)		2.69 ± 1.24	3.31 ± 1.32	0.601
P6 (skepticism)		5.05 ± 1.06	5.42 ± 1.36	0.131
P7 (hostility)		1.74 ± 1.04	2.04 ± 1.15	0.377
PANSS Negative score		20.45 ± 5.15	20.23 ± 5.87	0.406
PANSS General psychopathology		42.35 ± 7.40	42.43 ± 7.10	0.956
Hoffman score		24.69 ± 2.38	-	-

*The data are presented as the means ± standard deviation*.

*APG, Auditory hallucination patient group; NC, healthy controls; NPG, non-hallucinating patient group*.

a *P values were obtained by one-way analysis of variance tests*.

b *P value for gender distribution in the three groups was obtained by the chi-square test (P < 0.05)*.

* *The P values were obtained using two sample t-test (P < 0.05)*.

### MRI acquisition

A GEHDXT 3.0T Scanner was used to conduct all MRI scanning at the Radiology Department of Renmin Hospital of Wuhan University. High-resolution 3D T1-weighted structural imaging and resting-state fMRI scanning was performed for all patients. The following parameters were used for high-resolution 3D brain volume sequencing: TR/TE = 2000/30 ms; FOV = 220 mm × 220 mm; matrix = 64 × 64; FA = 90°; slice thickness = 1 mm; no gap in 188 sagittal slices. Resting-state fMRI data were generated with gradient-echo single-shot echo-planar imaging sequence with the following settings: TR/TE = 2000/30 ms; FOV = 220 mm × 220 mm; matrix = 64 × 64; FA = 90°; slice thickness = 4 mm; gap = 0.6 mm; 32 interleaved axial slices; and 240 volumes.

### Data preprocessing

MATLAB (MathWorks) was used for the preprocessing of generated data using the Data Processing Assistant for Resting-State fMRI (DPARSF) tool based upon Statistical Parametric Mapping (SPM8) and the Resting-State fMRI Data Analysis Toolkit(REST) (12). To eliminate any aberrant changes in the initial fMRI signal, the first five time points for each patient were omitted from analyses. Images were then corrected for head movement and slice trimming. Participants were only included in these analyses if they exhibited a maximum of 3 mm of displacement in the *x, y*, or *z* directions and no more than 3° of angular motion during scanning. The EPI template was then used to normalize functional images (voxel size: 3 × 3 × 3 mm^3^)(13), with the resultant normalized images being smoothed using a 3D isotropic Gaussian kernel (FWHM: 6 mm). Low-frequency drifts and high-frequency physiological noise were mitigated with a temporal filter (0.01–0.10 Hz). As recent evidence suggests that the effects of head motion can be more effectively eliminated when using higher-order models (14), mean frame-wise displacement (FD), which measures voxel-wise differences in motion and associated derivations, was assessed as a means of measuring head micromovements for study participants (15, 16). Nuisance regression was conducted through the use of cerebrospinal fluid (CSF), white matter, and global signals as covariates.

### Voxel-mirrored homotopic connectivity analyses

VMHC for each patient was measured based on the observed resting-state functional connectivity (rs-FC) among pairs of symmetric inter-hemispheric voxels. Briefly, VMHC maps were generated through Pearson correlation analyses of individual voxels and the mirror voxel in the opposite hemisphere of the brain (17). Measured correlation values were then subjected to Fisher z-transformation to improve associated normality, and group analyses were conducted with the resultant VMHC z-value data.

### Statistical analysis

Group comparisons were made by entering VMHC z-value maps into SPM8. Differences among the three participant groups were compared using one-way ANOVAs with groups as the between-subjects factor. Mean FD parameters including age, sex, education, illness duration, age of onset, and Chlorpromazine equivalents were used as covariates for group-level analyses. *P* < 0.001 was the significance threshold for group comparisons, with AlphaSim correction for multiple comparisons. *Post hoc* S-N-K *t*-tests were then used to assess sources of differences among groups in these analyses, with Pearson correlation analyses then being used to examine associations between VMHC results and patient symptoms.

## Results

### Study subject characteristics

Participants included in this group were age- and sex-matched, although patients in the NC group exhibited higher levels of educational attainment as compared to patients in the other groups. There were no significant differences between the two schizophrenia patient groups with respect to age, sex, level of education, duration of illness, average antipsychotic drug doses, or PANSS scores, although P3 (hallucination) scores did differ significantly between these groups (*P* < 0.01). The characteristics of all participating study subjects are compiled in Table 1.

### Differences in VMHC values among groups

**Figure 1:**
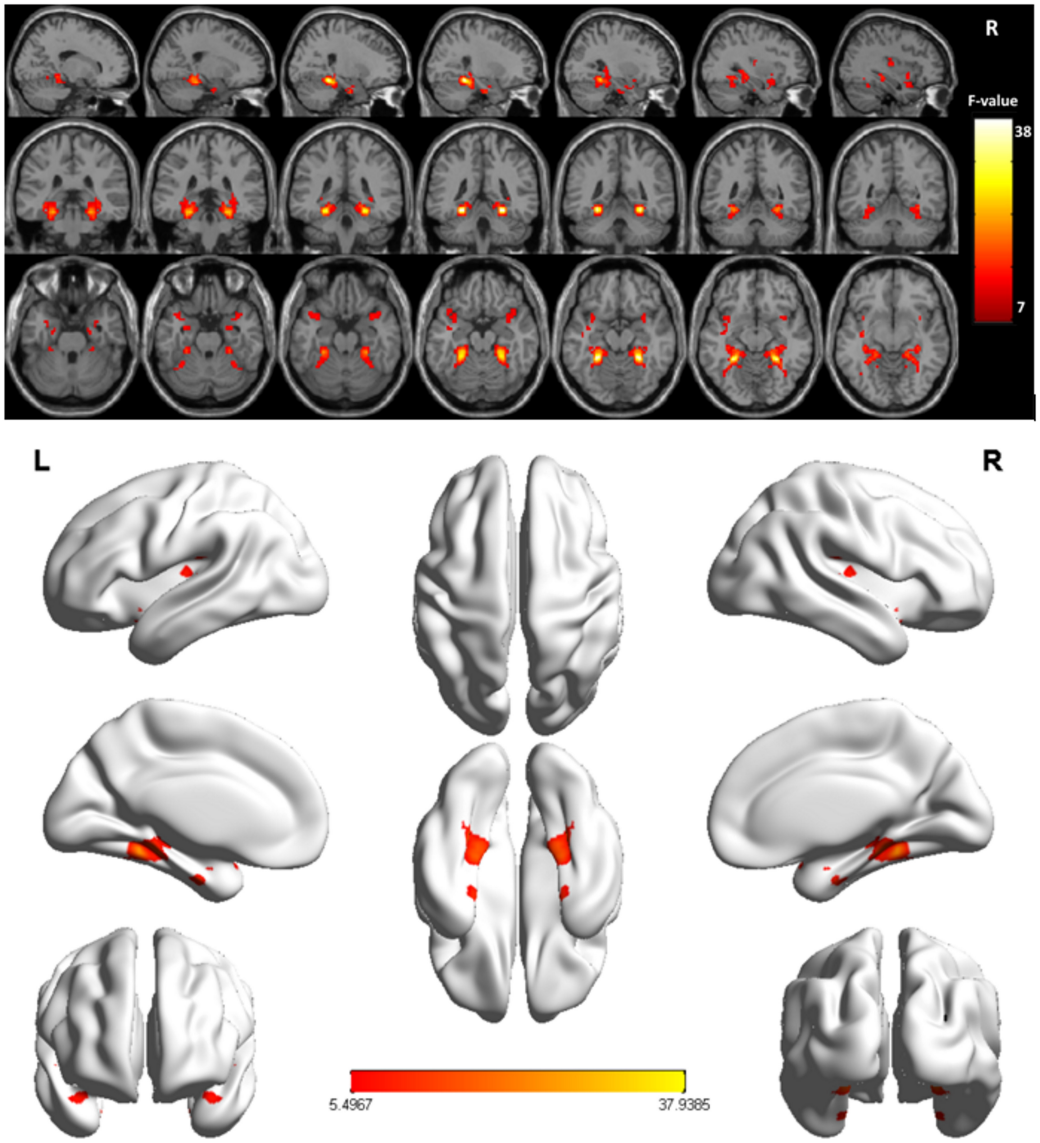
Significant differences of the VMHC values were fusiform gyrus extending into parahippocampus (fusiform cluster), STG extending into Heschl's gyrus, insula, and Rolandic operculum (STG cluster) across the three groups (NC, APG and NPG) (Corrected *p* < 0.005, cluster size = 52). Color bar indicates the F score.

Overall, VMHC values in the superior temporal gyrus (STG) extending into Heschl's gyrus, insula, and Rolandic operculum (collectively referred to as the STG cluster) and the fusiform gyrus extending into the parahippocampus (collectively referred to as the fusiform cluster) differed significantly among these three participant groups (Figure 1 and Table 2). *Post hoc t*-tests indicated that relative to individuals in the NC and NPG groups, patients in the APG group exhibited lower VMHC values in the fusiform cluster whereas no significant differences in this cluster were evident when comparing the NC and NPG groups. Additionally, reductions in STG cluster VMHC values were observed in both NPG and APG patients relative to NC individuals, although these changes were more pronounced for APG patients (Table 3).

**Table 2:** One-way ANOVA comparison on VMHC among three groups.

**Cluster location**	**Peak-MNI (X Y Z)**	**Voxel number**	**F**
Fusiform cluster	±24 −42 −15	80	37.94
STG cluster	±42 −24 21	68	5.50

**Table 3:** *Post hoc* pairwise comparisons of the mean VMHC z-score in three groups (^*^p < 0.05).

**Cluster location**	**NC**	**APG**	**NPG**	**F**	***Post-hoc t*** **-test**
Fusiform cluster	0.80 ± 0.18	0.57 ± 0.16	0.83 ± 0.12	37.94	NC /NPG > APG
STG cluster	0.87 ± 0.17	0.63 ± 0.17	0.73 ± 0.18	5.50	NC>NPG>APG

### Correlations between VMHC results and patient symptoms

**Figure 2:**
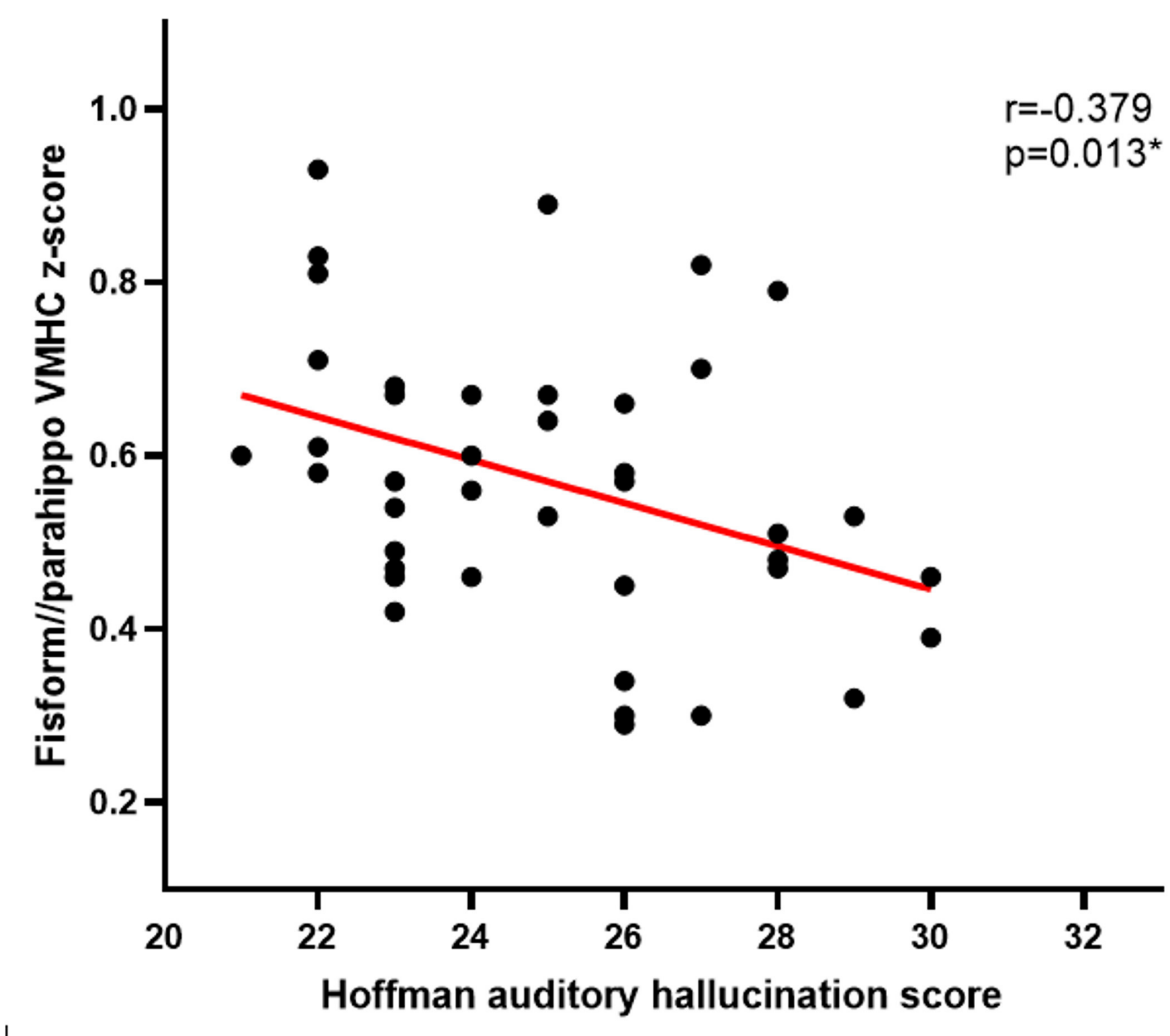
The Hoffman score was negatively correlated with the VMHC in the fusiform cluster in APG (*p* < 0.05).

**Figure 3:**
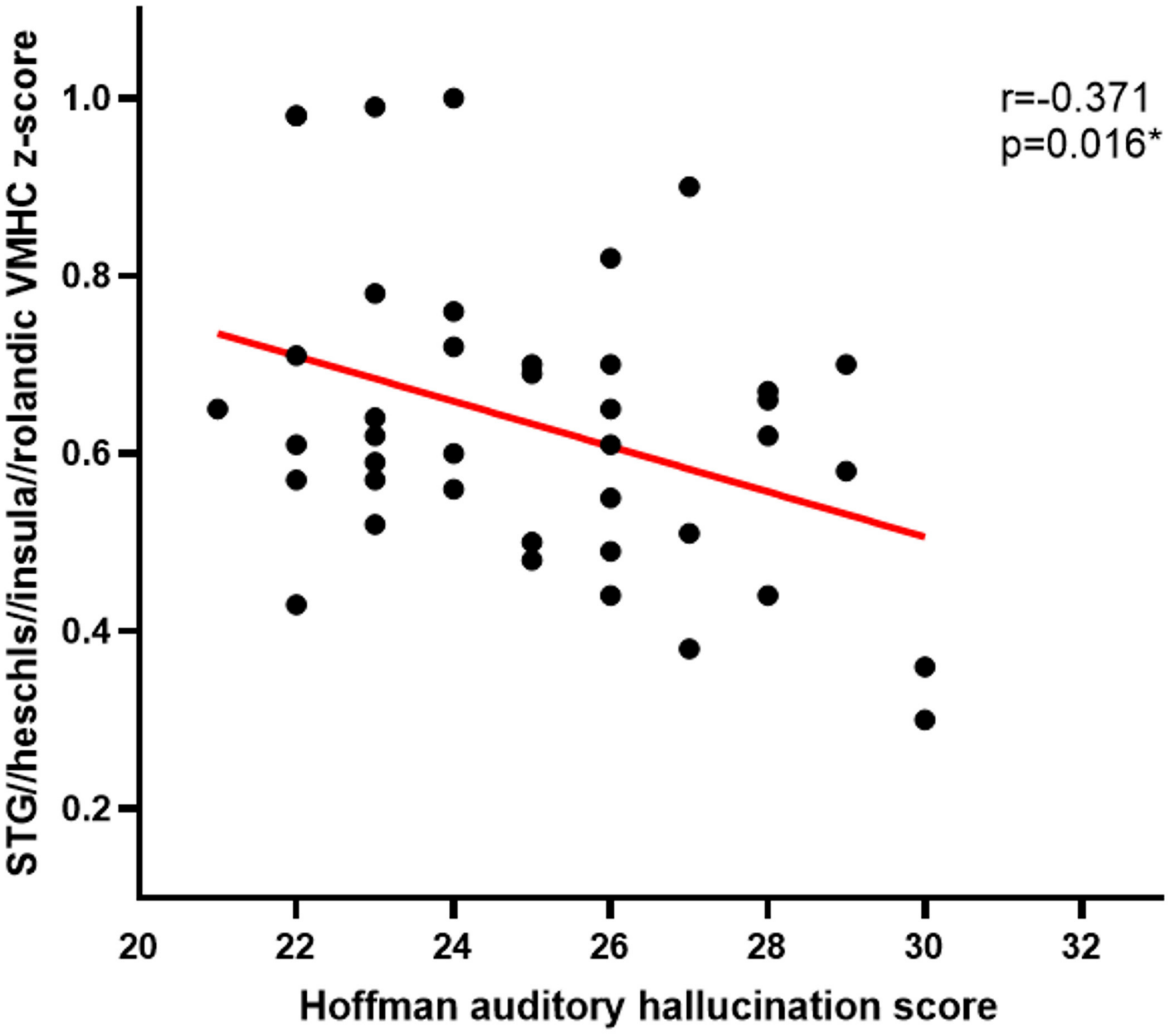
The Hoffman score was negatively correlated with the VMHC in the STG cluster in APG (*p* < 0.05).

Pearson correlation analyses revealed Hoffman scores and VMHC values were negatively correlated with one another in the fusiform cluster (r = −0.379, *P* = 0.013) (Figure 2) and the STG cluster (r = −0.371, *P* = 0.016) (Figure 3) among APG patients (*P* < 0.05).

## Discussion

The results of this study, which suggest that schizophrenia patients that experience AVHs exhibit significant changes in interhemispheric connectivity, offer robust neuroimaging support for the relationship between homotopic dysconnectivity and the occurrence of such hallucinations in schizophrenia (18). In contrast to prior studies, the present analysis was conducted after fully taking participant characteristics into consideration based on the assumption that other PANSS positive score items other than AVHs, such as delusions and suspicion, may have confounded the true relationship between AVH incidence and abnormal. As such, the comparable PANSS total score, PANSS negative score, PANSS positive score (with the exception of P3), and PANSS general psychopathology results in the APG and NPG groups in the present analysis effectively mitigated the potential confounding effects of these variables. Even after controlling for these factors, greater abnormal homotopic connectivity was still observed in schizophrenia patients affected by AVHs relative to patients not affected by these hallucinations.

The fusiform gyrus plays important roles in visual and sensory processing. When individuals experience abnormal sensory processing and aberrant voice recognition, this may lead to the false identification of one's own voice as emanating from an external source, contributing to the perception of AVHs (19). The observed aberrant homotopic connectivity of the fusiform gyrus in patients with AVHs in the present study cohort supported this conclusion. Notably, this abnormal homotopic connectivity of the fusiform cluster extended to the para-hippocampal gyrus in those patients that experienced AVHs. In a study conducted by Xiao et al., all analyzed schizophrenia patients similarly exhibited para-hippocampus abnormalities (18), in contrast to the results of the present study in which abnormalities in the para-hippocampus were only evident in AVH patients. VMHC values for this region of the brain were significantly negatively correlated with Hoffman scores, although more research will be necessary to explain this finding. The parahippocampus is an important component of the limbic system, which regulates memory storage and retrieval (20). Schizophrenia patients primarily report AVHs as consisting of critical speech hallucinations, and often report similar comments in their previous memories. Abnormal activity and connectivity of the parahippocampal gyrus may result in incorrect perceptions pertaining to the storage and extraction of information (8, 21). AVHs may thus develop as a consequence of the abnormal processing and retrieval of certain memories, as supported by a prior study in which patients experiencing hallucinations exhibited greater difficulty in determining the sources of memories (22).

With respect to the STG cluster, several studies have demonstrated that the role of the temporal lobe in voice recognition is inextricably linked to auditory hallucinations (23, 24). The more pronounced reduction in VMHC values in the STG and Heschl's gyrus in schizophrenia patients affected by AVHs further suggested that these regions are linked to the development of these hallucinations. Decreases in VMHC values in the STG and Heschl's gyrus would contribute to the dysfunctional cognition underlying AVHs through the incorrect identification of one's own voice as emanating from an external source. The Rolandic operculum is located in the frontal lobe and has repeatedly been shown to be closely linked to the mechanisms underlying psellism (7). One prior theoretical analysis of individuals with high schizotypy revealed alterations in both global properties and a reduction in the density of the gray matter in the Rolandic operculum in these patients (25). The Rolandic operculum also plays a role in the processing of auditory feedback, which may be linked to the processing of auditory feedback underlying AVH development (26). The insula is a multimodal convergence zone that plays a role in emotional regulation, contributing to the development of chronic positive symptoms together with the amygdala (27). One meta-analysis of functional data revealed a close relationship between AVHs in schizophrenia patients and the hippocampus, auditory cortex, and hippocampus (28). Consistently, structural and functional abnormalities may be suggestive of a central role for the insula in the production of AVHs (29).

## Conclusion

In conclusion, these results offer evidence in support of the homotopic disconnection hypothesis of schizophrenia, and are the first to suggest a potential role for reduced interhemispheric connectivity of the auditory and memory-related brain networks in the pathogenesis of AVHs following the exclusion of the effects of other confounding clinical symptoms on these results. In future analyses, additional participants will be recruited to conduct longitudinal analyses in which additional confounding factors will be excluded to minimize any potential bias.

## Data availability statement

The raw data supporting the conclusions of this article will be made available by the authors, without undue reservation.

## Ethics statement

The studies involving human participants were reviewed and approved by Renmin Hospital of Wuhan University. The patients/participants provided their written informed consent to participate in this study.

## Author contributions

CC, HW, and HH developed the initial idea for the manuscript. CC wrote the main body of the paper and including citations. HW and GW contributed to revision and editing of the manuscript. BR, LZ, and XQ analyzed the data. All authors contributed to approved the final manuscript.

## Funding

This research was supported by supported by the Fundamental Research Funds for the Central Universities (2042022kf1108) and the Medical Science Advancement Program of Wuhan University (TFLC2018001).

## Conflict of interest

The authors declare that the research was conducted in the absence of any commercial or financial relationships that could be construed as a potential conflict of interest.

## Publisher's note

All claims expressed in this article are solely those of the authors and do not necessarily represent those of their affiliated organizations, or those of the publisher, the editors and the reviewers. Any product that may be evaluated in this article, or claim that may be made by its manufacturer, is not guaranteed or endorsed by the publisher.
