# Supplementary material for: Reduced inter-hemispheric auditory and memory-related network interactions in patients with schizophrenia experiencing auditory verbal hallucinations
Source: Front Psychiatry. 2022 Aug 3;13:956895. doi: 10.3389/fpsyt.2022.956895 (PMC9381966; doi:10.3389/fpsyt.2022.956895)
Supplement: Supplementary file 1 [file Data_Sheet_1.PDF]

Excluding Criteria: 3.0mm and 3.0 degree in max head motion
NC0008
NC0011
NC0018
NC0024（数据质量有问题，已剔）
NC0025
NC0059
NC0089
NC0092
NC0093
NC1040
NC2015
SZ0008（数据质量有问题，已剔）
SZ0011
SZ0023
SZ0025
SZ0028（数据质量有问题，已剔）
SZ0044
SZ0048
SZ0057
SZ0088
SZ1049
SZ1061（数据质量有问题，已剔）
SZ1065（数据质量有问题，已剔）
SZ1066
SZ1069
SZ1070
SZ1075
SZ1077
SZ1083
SZ1095
SZ1099
SZ1104
SZ2012
SZ2015
SZ2025
SZ2029（数据质量有问题，已剔）
SZ2032
SZ2034
SZ2035

Excluding Criteria: 2.5mm and 2.5 degree in max head motion
NC0008
NC0011
NC0018
NC0024
NC0025
NC0026
NC0059
NC0073
NC0089
NC0092
NC0093
NC0105
NC1036
NC1040
NC1042
NC1051
NC1078
NC2015
SZ0001
SZ0006
SZ0008
SZ0011
SZ0023
SZ0025
SZ0028
SZ0044
SZ0046
SZ0048
SZ0057
SZ0077
SZ0088
SZ0102
SZ0115
SZ0132
SZ1049
SZ1052
SZ1057
SZ1061
SZ1063
SZ1065
SZ1066
SZ1069

SZ1070
SZ1075
SZ1077
SZ1083
SZ1095
SZ1099
SZ1103
SZ1104
SZ2012
SZ2015
SZ2025
SZ2029
SZ2032
SZ2034
SZ2035

Excluding Criteria: 2.0mm and 2.0 degree in max head motion
NC0001
NC0008
NC0011
NC0018
NC0024
NC0025
NC0026
NC0029
NC0033
NC0056
NC0059
NC0073
NC0077
NC0089
NC0092
NC0093
NC0095
NC0098
NC0105
NC1036
NC1040
NC1042
NC1050
NC1051

NC1054
NC1078
NC2012
NC2015
SZ0001
SZ0003
SZ0006
SZ0008
SZ0011
SZ0023
SZ0025
SZ0028
SZ0036
SZ0044
SZ0046
SZ0048
SZ0052
SZ0057
SZ0077
SZ0082
SZ0086
SZ0088
SZ0094
SZ0102
SZ0115
SZ0125
SZ0132
SZ1049
SZ1052
SZ1057
SZ1061
SZ1063
SZ1065
SZ1066
SZ1069
SZ1070
SZ1073
SZ1074
SZ1075
SZ1077
SZ1078
SZ1083
SZ1095
SZ1099

SZ1103
SZ1104
SZ2003
SZ2006
SZ2012
SZ2015
SZ2025
SZ2029
SZ2030
SZ2032
SZ2034
SZ2035

Excluding Criteria: 1.5mm and 1.5 degree in max head motion
NC0001
NC0008
NC0011
NC0013
NC0014
NC0018
NC0024
NC0025
NC0026
NC0028
NC0029
NC0033
NC0035
NC0040
NC0043
NC0046
NC0052
NC0056
NC0059
NC0073
NC0077
NC0089
NC0092
NC0093
NC0094
NC0095
NC0096

NC0097
NC0098
NC0105
NC1036
NC1040
NC1041
NC1042
NC1050
NC1051
NC1054
NC1078
NC2003
NC2012
NC2015
SZ0001
SZ0003
SZ0006
SZ0007
SZ0008
SZ0010
SZ0011
SZ0023
SZ0025
SZ0028
SZ0029
SZ0036
SZ0044
SZ0046
SZ0048
SZ0052
SZ0053
SZ0057
SZ0071
SZ0077
SZ0082
SZ0086
SZ0088
SZ0094
SZ0097
SZ0102
SZ0109
SZ0113
SZ0115
SZ0118

SZ0125
SZ0132
SZ1049
SZ1052
SZ1053
SZ1055
SZ1057
SZ1061
SZ1063
SZ1064
SZ1065
SZ1066
SZ1067
SZ1069
SZ1070
SZ1073
SZ1074
SZ1075
SZ1077
SZ1078
SZ1083
SZ1086
SZ1094
SZ1095
SZ1099
SZ1103
SZ1104
SZ2003
SZ2006
SZ2012
SZ2015
SZ2023
SZ2025
SZ2029
SZ2030
SZ2031
SZ2032
SZ2034
SZ2035

Excluding Criteria: 1.0mm and 1.0 degree in max head motion

NC0001
NC0008
NC0011
NC0013
NC0014
NC0018
NC0023
NC0024
NC0025
NC0026
NC0028
NC0029
NC0032
NC0033
NC0035
NC0040
NC0043
NC0046
NC0052
NC0054
NC0056
NC0057
NC0059
NC0060
NC0063
NC0068
NC0069
NC0071
NC0073
NC0074
NC0075
NC0076
NC0077
NC0088
NC0089
NC0090
NC0092
NC0093
NC0094
NC0095
NC0096
NC0097
NC0098
NC0099

NC0100
NC0105
NC0107
NC1035
NC1036
NC1039
NC1040
NC1041
NC1042
NC1043
NC1045
NC1046
NC1049
NC1050
NC1051
NC1054
NC1055
NC1056
NC1065
NC1070
NC1078
NC1080
NC2003
NC2005
NC2010
NC2012
NC2015
NC2021
SZ0001
SZ0003
SZ0006
SZ0007
SZ0008
SZ0010
SZ0011
SZ0017
SZ0019
SZ0023
SZ0025
SZ0028
SZ0029
SZ0031
SZ0032
SZ0036

SZ0043
SZ0044
SZ0046
SZ0048
SZ0052
SZ0053
SZ0057
SZ0062
SZ0066
SZ0067
SZ0071
SZ0077
SZ0080
SZ0082
SZ0086
SZ0088
SZ0093
SZ0094
SZ0095
SZ0096
SZ0097
SZ0099
SZ0102
SZ0105
SZ0109
SZ0110
SZ0111
SZ0113
SZ0115
SZ0118
SZ0125
SZ0132
SZ1044
SZ1049
SZ1050
SZ1052
SZ1053
SZ1054
SZ1055
SZ1057
SZ1061
SZ1063
SZ1064
SZ1065

SZ1066
SZ1067
SZ1069
SZ1070
SZ1073
SZ1074
SZ1075
SZ1076
SZ1077
SZ1078
SZ1080
SZ1082
SZ1083
SZ1086
SZ1090
SZ1092
SZ1094
SZ1095
SZ1098
SZ1099
SZ1100
SZ1101
SZ1103
SZ1104
SZ2003
SZ2004
SZ2006
SZ2009
SZ2012
SZ2015
SZ2023
SZ2025
SZ2028
SZ2029
SZ2030
SZ2031
SZ2032
SZ2034
SZ2035

Excluding Criteria: 0.5mm and 0.5 degree in max head motion

NC0001
NC0004
NC0005
NC0007
NC0008
NC0009
NC0010
NC0011
NC0013
NC0014
NC0015
NC0016
NC0018
NC0020
NC0023
NC0024
NC0025
NC0026
NC0027
NC0028
NC0029
NC0030
NC0031
NC0032
NC0033
NC0035
NC0036
NC0038
NC0039
NC0040
NC0042
NC0043
NC0044
NC0045
NC0046
NC0048
NC0050
NC0051
NC0052
NC0054
NC0056
NC0057
NC0058
NC0059

NC0060
NC0061
NC0063
NC0064
NC0065
NC0066
NC0067
NC0068
NC0069
NC0070
NC0071
NC0073
NC0074
NC0075
NC0076
NC0077
NC0078
NC0079
NC0080
NC0081
NC0083
NC0084
NC0087
NC0088
NC0089
NC0090
NC0092
NC0093
NC0094
NC0095
NC0096
NC0097
NC0098
NC0099
NC0100
NC0102
NC0103
NC0104
NC0105
NC0107
NC0108
NC1035
NC1036
NC1039

NC1040
NC1041
NC1042
NC1043
NC1044
NC1045
NC1046
NC1048
NC1049
NC1050
NC1051
NC1052
NC1053
NC1054
NC1055
NC1056
NC1057
NC1058
NC1059
NC1060
NC1062
NC1065
NC1069
NC1070
NC1073
NC1075
NC1076
NC1078
NC1079
NC1080
NC1081
NC2001
NC2003
NC2005
NC2008
NC2009
NC2010
NC2011
NC2012
NC2013
NC2015
NC2016
NC2017
NC2018

NC2019
NC2021
NC2022
NC2023
NC2024
SZ0001
SZ0003
SZ0006
SZ0007
SZ0008
SZ0009
SZ0010
SZ0011
SZ0012
SZ0015
SZ0016
SZ0017
SZ0018
SZ0019
SZ0022
SZ0023
SZ0025
SZ0027
SZ0028
SZ0029
SZ0031
SZ0032
SZ0036
SZ0037
SZ0038
SZ0043
SZ0044
SZ0046
SZ0048
SZ0049
SZ0050
SZ0051
SZ0052
SZ0053
SZ0054
SZ0056
SZ0057
SZ0059
SZ0061

SZ0062
SZ0064
SZ0065
SZ0066
SZ0067
SZ0069
SZ0070
SZ0071
SZ0073
SZ0076
SZ0077
SZ0079
SZ0080
SZ0082
SZ0085
SZ0086
SZ0087
SZ0088
SZ0089
SZ0091
SZ0092
SZ0093
SZ0094
SZ0095
SZ0096
SZ0097
SZ0098
SZ0099
SZ0100
SZ0102
SZ0103
SZ0105
SZ0107
SZ0109
SZ0110
SZ0111
SZ0112
SZ0113
SZ0115
SZ0118
SZ0119
SZ0123
SZ0124
SZ0125

SZ0127
SZ0128
SZ0129
SZ0130
SZ0131
SZ0132
SZ0133
SZ0135
SZ1044
SZ1049
SZ1050
SZ1052
SZ1053
SZ1054
SZ1055
SZ1056
SZ1057
SZ1058
SZ1059
SZ1060
SZ1061
SZ1062
SZ1063
SZ1064
SZ1065
SZ1066
SZ1067
SZ1068
SZ1069
SZ1070
SZ1071
SZ1073
SZ1074
SZ1075
SZ1076
SZ1077
SZ1078
SZ1080
SZ1081
SZ1082
SZ1083
SZ1086
SZ1087
SZ1088

SZ1090
SZ1092
SZ1093
SZ1094
SZ1095
SZ1096
SZ1098
SZ1099
SZ1100
SZ1101
SZ1103
SZ1104
SZ2001
SZ2003
SZ2004
SZ2005
SZ2006
SZ2007
SZ2008
SZ2009
SZ2010
SZ2012
SZ2015
SZ2022
SZ2023
SZ2024
SZ2025
SZ2027
SZ2028
SZ2029
SZ2030
SZ2031
SZ2032
SZ2033
SZ2034
SZ2035
SZ2036
SZ2037

Excluding Criteria: 3.0mm and 3.0 degree in max head motion
NC1039

SZ1095
SZ1099

Excluding Criteria: 2.5mm and 2.5 degree in max head motion
NC1039
SZ1063
SZ1095
SZ1099
SZ1103

Excluding Criteria: 2.0mm and 2.0 degree in max head motion
NC1039
SZ1063
SZ1095
SZ1099
SZ1103

Excluding Criteria: 1.5mm and 1.5 degree in max head motion
NC1039
SZ1063
SZ1095
SZ1099
SZ1103

Excluding Criteria: 1.0mm and 1.0 degree in max head motion
NC1038
NC1039
SZ0099
SZ1063
SZ1092
SZ1095
SZ1099

SZ1100
SZ1103

Excluding Criteria: 0.5mm and 0.5 degree in max head motion
NC1038
NC1039
SZ0099
SZ1063
SZ1092
SZ1093
SZ1095
SZ1099
SZ1100
SZ1103

Excluding Criteria: 3.0mm and 3.0 degree in max head motion
NC1039
SZ1095
SZ1099

Excluding Criteria: 2.5mm and 2.5 degree in max head motion
NC1039
SZ1063
SZ1095
SZ1099
SZ1103

Excluding Criteria: 2.0mm and 2.0 degree in max head motion
NC1039
SZ1063
SZ1095
SZ1099

SZ1103

Excluding Criteria: 1.5mm and 1.5 degree in max head motion
NC1039
SZ1063
SZ1095
SZ1099
SZ1103

Excluding Criteria: 1.0mm and 1.0 degree in max head motion
NC1038
NC1039
SZ0099
SZ1063
SZ1092
SZ1095
SZ1099
SZ1100
SZ1103

Excluding Criteria: 0.5mm and 0.5 degree in max head motion
NC1038
NC1039
SZ0099
SZ1063
SZ1092
SZ1093
SZ1095
SZ1099
SZ1100
SZ1103

Excluding Criteria: 3.0mm and 3.0 degree in max head motion
NC1039
SZ1095
SZ1099

Excluding Criteria: 2.5mm and 2.5 degree in max head motion
NC1039
SZ1063
SZ1095
SZ1099
SZ1103

Excluding Criteria: 2.0mm and 2.0 degree in max head motion
NC1039
SZ1063
SZ1095
SZ1099
SZ1103

Excluding Criteria: 1.5mm and 1.5 degree in max head motion
NC1039
SZ1063
SZ1095
SZ1099
SZ1103

Excluding Criteria: 1.0mm and 1.0 degree in max head motion
NC1038
NC1039
SZ0099
SZ1063
SZ1092

SZ1095
SZ1099
SZ1100
SZ1103

Excluding Criteria: 0.5mm and 0.5 degree in max head motion
NC1038
NC1039
SZ0099
SZ1063
SZ1092
SZ1093
SZ1095
SZ1099
SZ1100
SZ1103

Excluding Criteria: 3.0mm and 3.0 degree in max head motion
SZ1095
SZ1099

Excluding Criteria: 2.5mm and 2.5 degree in max head motion
SZ1063
SZ1095
SZ1099
SZ1103

Excluding Criteria: 2.0mm and 2.0 degree in max head motion
SZ1063
SZ1095
SZ1099
SZ1103

Excluding Criteria: 1.5mm and 1.5 degree in max head motion
SZ1063
SZ1095
SZ1099
SZ1103

Excluding Criteria: 1.0mm and 1.0 degree in max head motion
NC1038
SZ0099
SZ1063
SZ1092
SZ1095
SZ1099
SZ1100
SZ1103

Excluding Criteria: 0.5mm and 0.5 degree in max head motion
NC1038
NC1039
SZ0099
SZ1063
SZ1092
SZ1093
SZ1095
SZ1099
SZ1100
SZ1103

Excluding Criteria: 3.0mm and 3.0 degree in max head motion
NC0008
NC0011

NC0018
NC0024
NC0025
NC0059
NC0089
NC0092
NC0093
NC1039
NC1040
NC2015
SZ0008
SZ0011
SZ0023
SZ0025
SZ0028
SZ0044
SZ0048
SZ0057
SZ0088
SZ1049
SZ1061
SZ1065
SZ1066
SZ1069
SZ1070
SZ1075
SZ1077
SZ1083
SZ1095
SZ1099
SZ1104
SZ2012
SZ2015
SZ2025
SZ2029
SZ2032
SZ2034
SZ2035

Excluding Criteria: 2.5mm and 2.5 degree in max head motion
NC0008

NC0011
NC0018
NC0024
NC0025
NC0026
NC0059
NC0073
NC0089
NC0092
NC0093
NC0105
NC1036
NC1039
NC1040
NC1042
NC1051
NC1078
NC2015
SZ0001
SZ0006
SZ0008
SZ0011
SZ0023
SZ0025
SZ0028
SZ0044
SZ0046
SZ0048
SZ0057
SZ0077
SZ0088
SZ0102
SZ0115
SZ0132
SZ1049
SZ1052
SZ1057
SZ1061
SZ1063
SZ1065
SZ1066
SZ1069
SZ1070
SZ1075

SZ1077
SZ1083
SZ1095
SZ1099
SZ1103
SZ1104
SZ2012
SZ2015
SZ2025
SZ2029
SZ2032
SZ2034
SZ2035

Excluding Criteria: 2.0mm and 2.0 degree in max head motion
NC0001
NC0008
NC0011
NC0018
NC0024
NC0025
NC0026
NC0029
NC0033
NC0056
NC0059
NC0073
NC0077
NC0089
NC0092
NC0093
NC0095
NC0098
NC0105
NC1036
NC1039
NC1040
NC1042
NC1050
NC1051
NC1054

NC1078
NC2012
NC2015
SZ0001
SZ0003
SZ0006
SZ0008
SZ0011
SZ0023
SZ0025
SZ0028
SZ0036
SZ0044
SZ0046
SZ0048
SZ0052
SZ0057
SZ0077
SZ0082
SZ0086
SZ0088
SZ0094
SZ0102
SZ0115
SZ0125
SZ0132
SZ1049
SZ1052
SZ1057
SZ1061
SZ1063
SZ1065
SZ1066
SZ1069
SZ1070
SZ1073
SZ1074
SZ1075
SZ1077
SZ1078
SZ1083
SZ1095
SZ1099
SZ1103

SZ1104
SZ2003
SZ2006
SZ2012
SZ2015
SZ2025
SZ2029
SZ2030
SZ2032
SZ2034
SZ2035

Excluding Criteria: 1.5mm and 1.5 degree in max head motion
NC0001
NC0008
NC0011
NC0013
NC0014
NC0018
NC0024
NC0025
NC0026
NC0028
NC0029
NC0033
NC0035
NC0040
NC0043
NC0046
NC0052
NC0056
NC0059
NC0073
NC0077
NC0089
NC0092
NC0093
NC0094
NC0095
NC0096
NC0097

NC0098
NC0105
NC1036
NC1039
NC1040
NC1041
NC1042
NC1050
NC1051
NC1054
NC1078
NC2003
NC2012
NC2015
SZ0001
SZ0003
SZ0006
SZ0007
SZ0008
SZ0010
SZ0011
SZ0023
SZ0025
SZ0028
SZ0029
SZ0036
SZ0044
SZ0046
SZ0048
SZ0052
SZ0053
SZ0057
SZ0071
SZ0077
SZ0082
SZ0086
SZ0088
SZ0094
SZ0097
SZ0102
SZ0109
SZ0113
SZ0115
SZ0118

SZ0125
SZ0132
SZ1049
SZ1052
SZ1053
SZ1055
SZ1057
SZ1061
SZ1063
SZ1064
SZ1065
SZ1066
SZ1067
SZ1069
SZ1070
SZ1073
SZ1074
SZ1075
SZ1077
SZ1078
SZ1083
SZ1086
SZ1094
SZ1095
SZ1099
SZ1103
SZ1104
SZ2003
SZ2006
SZ2012
SZ2015
SZ2023
SZ2025
SZ2029
SZ2030
SZ2031
SZ2032
SZ2034
SZ2035

Excluding Criteria: 1.0mm and 1.0 degree in max head motion

NC0001
NC0008
NC0011
NC0013
NC0014
NC0018
NC0023
NC0024
NC0025
NC0026
NC0028
NC0029
NC0032
NC0033
NC0035
NC0040
NC0043
NC0046
NC0052
NC0054
NC0056
NC0057
NC0059
NC0060
NC0063
NC0068
NC0069
NC0071
NC0073
NC0074
NC0075
NC0076
NC0077
NC0088
NC0089
NC0090
NC0092
NC0093
NC0094
NC0095
NC0096
NC0097
NC0098
NC0099

NC0100
NC0105
NC0107
NC1035
NC1036
NC1038
NC1039
NC1040
NC1041
NC1042
NC1043
NC1045
NC1046
NC1049
NC1050
NC1051
NC1054
NC1055
NC1056
NC1065
NC1070
NC1078
NC1080
NC2003
NC2005
NC2010
NC2012
NC2015
NC2021
SZ0001
SZ0003
SZ0006
SZ0007
SZ0008
SZ0010
SZ0011
SZ0017
SZ0019
SZ0023
SZ0025
SZ0028
SZ0029
SZ0031
SZ0032

SZ0036
SZ0043
SZ0044
SZ0046
SZ0048
SZ0052
SZ0053
SZ0057
SZ0062
SZ0066
SZ0067
SZ0071
SZ0077
SZ0080
SZ0082
SZ0086
SZ0088
SZ0093
SZ0094
SZ0095
SZ0096
SZ0097
SZ0099
SZ0102
SZ0105
SZ0109
SZ0110
SZ0111
SZ0113
SZ0115
SZ0118
SZ0125
SZ0132
SZ1044
SZ1049
SZ1050
SZ1052
SZ1053
SZ1054
SZ1055
SZ1057
SZ1061
SZ1063
SZ1064

SZ1065
SZ1066
SZ1067
SZ1069
SZ1070
SZ1073
SZ1074
SZ1075
SZ1076
SZ1077
SZ1078
SZ1080
SZ1082
SZ1083
SZ1086
SZ1090
SZ1092
SZ1094
SZ1095
SZ1098
SZ1099
SZ1100
SZ1103
SZ1104
SZ2003
SZ2004
SZ2006
SZ2009
SZ2012
SZ2015
SZ2023
SZ2025
SZ2028
SZ2029
SZ2030
SZ2031
SZ2032
SZ2034
SZ2035

Excluding Criteria: 0.5mm and 0.5 degree in max head motion

NC0001
NC0004
NC0005
NC0007
NC0008
NC0009
NC0010
NC0011
NC0013
NC0014
NC0015
NC0016
NC0018
NC0020
NC0023
NC0024
NC0025
NC0026
NC0027
NC0028
NC0029
NC0030
NC0031
NC0032
NC0033
NC0035
NC0036
NC0038
NC0039
NC0040
NC0042
NC0043
NC0044
NC0045
NC0046
NC0048
NC0050
NC0051
NC0052
NC0054
NC0056
NC0057
NC0058
NC0059

NC0060
NC0061
NC0063
NC0064
NC0065
NC0066
NC0067
NC0068
NC0069
NC0070
NC0071
NC0073
NC0074
NC0075
NC0076
NC0077
NC0078
NC0079
NC0080
NC0081
NC0083
NC0084
NC0087
NC0088
NC0089
NC0090
NC0092
NC0093
NC0094
NC0095
NC0096
NC0097
NC0098
NC0099
NC0100
NC0102
NC0103
NC0104
NC0105
NC0107
NC0108
NC1035
NC1036
NC1038

NC1039
NC1040
NC1041
NC1042
NC1043
NC1044
NC1045
NC1046
NC1048
NC1049
NC1050
NC1051
NC1052
NC1053
NC1054
NC1055
NC1056
NC1057
NC1058
NC1059
NC1060
NC1062
NC1065
NC1069
NC1070
NC1073
NC1075
NC1076
NC1078
NC1079
NC1080
NC1081
NC2001
NC2003
NC2005
NC2008
NC2009
NC2010
NC2011
NC2012
NC2013
NC2015
NC2016
NC2017

NC2018
NC2019
NC2021
NC2022
NC2023
NC2024
SZ0001
SZ0003
SZ0006
SZ0007
SZ0008
SZ0009
SZ0010
SZ0011
SZ0012
SZ0015
SZ0016
SZ0017
SZ0018
SZ0019
SZ0022
SZ0023
SZ0025
SZ0027
SZ0028
SZ0029
SZ0031
SZ0032
SZ0036
SZ0037
SZ0038
SZ0043
SZ0044
SZ0046
SZ0048
SZ0049
SZ0050
SZ0051
SZ0052
SZ0053
SZ0054
SZ0056
SZ0057
SZ0059

SZ0061
SZ0062
SZ0064
SZ0065
SZ0066
SZ0067
SZ0069
SZ0070
SZ0071
SZ0073
SZ0076
SZ0077
SZ0079
SZ0080
SZ0082
SZ0085
SZ0086
SZ0087
SZ0088
SZ0089
SZ0091
SZ0092
SZ0093
SZ0094
SZ0095
SZ0096
SZ0097
SZ0098
SZ0099
SZ0100
SZ0102
SZ0103
SZ0105
SZ0107
SZ0109
SZ0110
SZ0111
SZ0112
SZ0113
SZ0115
SZ0118
SZ0119
SZ0123
SZ0124

SZ0125
SZ0127
SZ0128
SZ0129
SZ0130
SZ0131
SZ0132
SZ0133
SZ0135
SZ1044
SZ1049
SZ1050
SZ1052
SZ1053
SZ1054
SZ1055
SZ1056
SZ1057
SZ1058
SZ1059
SZ1060
SZ1061
SZ1062
SZ1063
SZ1064
SZ1065
SZ1066
SZ1067
SZ1068
SZ1069
SZ1070
SZ1071
SZ1073
SZ1074
SZ1075
SZ1076
SZ1077
SZ1078
SZ1080
SZ1081
SZ1082
SZ1083
SZ1086
SZ1087

SZ1088
SZ1090
SZ1092
SZ1093
SZ1094
SZ1095
SZ1096
SZ1098
SZ1099
SZ1100
SZ1103
SZ1104
SZ2001
SZ2003
SZ2004
SZ2005
SZ2006
SZ2007
SZ2008
SZ2009
SZ2010
SZ2012
SZ2015
SZ2022
SZ2023
SZ2024
SZ2025
SZ2027
SZ2028
SZ2029
SZ2030
SZ2031
SZ2032
SZ2033
SZ2034
SZ2035
SZ2036
SZ2037

Excluding Criteria: 3.0mm and 3.0 degree in max head motion
NC0008

NC0011
NC0018
NC0024
NC0025
NC0059
NC0089
NC0092
NC0093
NC1040
NC2015
SZ0008
SZ0011
SZ0023
SZ0025
SZ0028
SZ0044
SZ0048
SZ0057
SZ0088
SZ1049
SZ1061
SZ1065
SZ1066
SZ1069
SZ1070
SZ1075
SZ1077
SZ1083
SZ1095
SZ1099
SZ1104
SZ2012
SZ2015
SZ2025
SZ2029
SZ2032
SZ2034
SZ2035

Excluding Criteria: 2.5mm and 2.5 degree in max head motion
NC0008

NC0011
NC0018
NC0024
NC0025
NC0026
NC0059
NC0073
NC0089
NC0092
NC0093
NC0105
NC1036
NC1040
NC1042
NC1051
NC1078
NC2015
SZ0001
SZ0006
SZ0008
SZ0011
SZ0023
SZ0025
SZ0028
SZ0044
SZ0046
SZ0048
SZ0057
SZ0077
SZ0088
SZ0102
SZ0115
SZ0132
SZ1049
SZ1052
SZ1057
SZ1061
SZ1063
SZ1065
SZ1066
SZ1069
SZ1070
SZ1075
SZ1077

SZ1083
SZ1095
SZ1099
SZ1103
SZ1104
SZ2012
SZ2015
SZ2025
SZ2029
SZ2032
SZ2034
SZ2035

Excluding Criteria: 2.0mm and 2.0 degree in max head motion
NC0001
NC0008
NC0011
NC0018
NC0024
NC0025
NC0026
NC0029
NC0033
NC0056
NC0059
NC0073
NC0077
NC0089
NC0092
NC0093
NC0095
NC0098
NC0105
NC1036
NC1040
NC1042
NC1050
NC1051
NC1054
NC1078
NC2012

NC2015
SZ0001
SZ0003
SZ0006
SZ0008
SZ0011
SZ0023
SZ0025
SZ0028
SZ0036
SZ0044
SZ0046
SZ0048
SZ0052
SZ0057
SZ0077
SZ0082
SZ0086
SZ0088
SZ0094
SZ0102
SZ0115
SZ0125
SZ0132
SZ1049
SZ1052
SZ1057
SZ1061
SZ1063
SZ1065
SZ1066
SZ1069
SZ1070
SZ1073
SZ1074
SZ1075
SZ1077
SZ1078
SZ1083
SZ1095
SZ1099
SZ1103
SZ1104
SZ2003

SZ2006
SZ2012
SZ2015
SZ2025
SZ2029
SZ2030
SZ2032
SZ2034
SZ2035

Excluding Criteria: 1.5mm and 1.5 degree in max head motion
NC0001
NC0008
NC0011
NC0013
NC0014
NC0018
NC0024
NC0025
NC0026
NC0028
NC0029
NC0033
NC0035
NC0040
NC0043
NC0046
NC0052
NC0056
NC0059
NC0073
NC0077
NC0089
NC0092
NC0093
NC0094
NC0095
NC0096
NC0097
NC0098
NC0105

NC1036
NC1040
NC1041
NC1042
NC1050
NC1051
NC1054
NC1078
NC2003
NC2012
NC2015
SZ0001
SZ0003
SZ0006
SZ0007
SZ0008
SZ0010
SZ0011
SZ0023
SZ0025
SZ0028
SZ0029
SZ0036
SZ0044
SZ0046
SZ0048
SZ0052
SZ0053
SZ0057
SZ0071
SZ0077
SZ0082
SZ0086
SZ0088
SZ0094
SZ0097
SZ0102
SZ0109
SZ0113
SZ0115
SZ0118
SZ0125
SZ0132
SZ1049

SZ1052
SZ1053
SZ1055
SZ1057
SZ1061
SZ1063
SZ1064
SZ1065
SZ1066
SZ1067
SZ1069
SZ1070
SZ1073
SZ1074
SZ1075
SZ1077
SZ1078
SZ1083
SZ1086
SZ1094
SZ1095
SZ1099
SZ1103
SZ1104
SZ2003
SZ2006
SZ2012
SZ2015
SZ2023
SZ2025
SZ2029
SZ2030
SZ2031
SZ2032
SZ2034
SZ2035

Excluding Criteria: 1.0mm and 1.0 degree in max head motion
NC0001
NC0008
NC0011

NC0013
NC0014
NC0018
NC0023
NC0024
NC0025
NC0026
NC0028
NC0029
NC0032
NC0033
NC0035
NC0040
NC0043
NC0046
NC0052
NC0054
NC0056
NC0057
NC0059
NC0060
NC0063
NC0068
NC0069
NC0071
NC0073
NC0074
NC0075
NC0076
NC0077
NC0088
NC0089
NC0090
NC0092
NC0093
NC0094
NC0095
NC0096
NC0097
NC0098
NC0099
NC0100
NC0105
NC0107

NC1035
NC1036
NC1038
NC1039
NC1040
NC1041
NC1042
NC1043
NC1045
NC1046
NC1049
NC1050
NC1051
NC1054
NC1055
NC1056
NC1065
NC1070
NC1078
NC1080
NC2003
NC2005
NC2010
NC2012
NC2015
NC2021
SZ0001
SZ0003
SZ0006
SZ0007
SZ0008
SZ0010
SZ0011
SZ0017
SZ0019
SZ0023
SZ0025
SZ0028
SZ0029
SZ0031
SZ0032
SZ0036
SZ0043
SZ0044

SZ0046
SZ0048
SZ0052
SZ0053
SZ0057
SZ0062
SZ0066
SZ0067
SZ0071
SZ0077
SZ0080
SZ0082
SZ0086
SZ0088
SZ0093
SZ0094
SZ0095
SZ0096
SZ0097
SZ0099
SZ0102
SZ0105
SZ0109
SZ0110
SZ0111
SZ0113
SZ0115
SZ0118
SZ0125
SZ0132
SZ1044
SZ1049
SZ1050
SZ1052
SZ1053
SZ1054
SZ1055
SZ1057
SZ1061
SZ1063
SZ1064
SZ1065
SZ1066
SZ1067

SZ1069
SZ1070
SZ1073
SZ1074
SZ1075
SZ1076
SZ1077
SZ1078
SZ1080
SZ1082
SZ1083
SZ1086
SZ1090
SZ1092
SZ1094
SZ1095
SZ1098
SZ1099
SZ1100
SZ1103
SZ1104
SZ2003
SZ2004
SZ2006
SZ2009
SZ2012
SZ2015
SZ2023
SZ2025
SZ2028
SZ2029
SZ2030
SZ2031
SZ2032
SZ2034
SZ2035

Excluding Criteria: 0.5mm and 0.5 degree in max head motion
NC0001
NC0004
NC0005

NC0007
NC0008
NC0009
NC0010
NC0011
NC0013
NC0014
NC0015
NC0016
NC0018
NC0020
NC0023
NC0024
NC0025
NC0026
NC0027
NC0028
NC0029
NC0030
NC0031
NC0032
NC0033
NC0035
NC0036
NC0038
NC0039
NC0040
NC0042
NC0043
NC0044
NC0045
NC0046
NC0048
NC0050
NC0051
NC0052
NC0054
NC0056
NC0057
NC0058
NC0059
NC0060
NC0061
NC0063

NC0064
NC0065
NC0066
NC0067
NC0068
NC0069
NC0070
NC0071
NC0073
NC0074
NC0075
NC0076
NC0077
NC0078
NC0079
NC0080
NC0081
NC0083
NC0084
NC0087
NC0088
NC0089
NC0090
NC0092
NC0093
NC0094
NC0095
NC0096
NC0097
NC0098
NC0099
NC0100
NC0102
NC0103
NC0104
NC0105
NC0107
NC0108
NC1035
NC1036
NC1038
NC1039
NC1040
NC1041

NC1042
NC1043
NC1044
NC1045
NC1046
NC1048
NC1049
NC1050
NC1051
NC1052
NC1053
NC1054
NC1055
NC1056
NC1057
NC1058
NC1059
NC1060
NC1062
NC1065
NC1069
NC1070
NC1073
NC1075
NC1076
NC1078
NC1079
NC1080
NC1081
NC2001
NC2003
NC2005
NC2008
NC2009
NC2010
NC2011
NC2012
NC2013
NC2015
NC2016
NC2017
NC2018
NC2019
NC2021

NC2022
NC2023
NC2024
SZ0001
SZ0003
SZ0006
SZ0007
SZ0008
SZ0009
SZ0010
SZ0011
SZ0012
SZ0015
SZ0016
SZ0017
SZ0018
SZ0019
SZ0022
SZ0023
SZ0025
SZ0027
SZ0028
SZ0029
SZ0031
SZ0032
SZ0036
SZ0037
SZ0038
SZ0043
SZ0044
SZ0046
SZ0048
SZ0049
SZ0050
SZ0051
SZ0052
SZ0053
SZ0054
SZ0056
SZ0057
SZ0059
SZ0061
SZ0062
SZ0064

SZ0065
SZ0066
SZ0067
SZ0069
SZ0070
SZ0071
SZ0073
SZ0076
SZ0077
SZ0079
SZ0080
SZ0082
SZ0085
SZ0086
SZ0087
SZ0088
SZ0089
SZ0091
SZ0092
SZ0093
SZ0094
SZ0095
SZ0096
SZ0097
SZ0098
SZ0099
SZ0100
SZ0102
SZ0103
SZ0105
SZ0107
SZ0109
SZ0110
SZ0111
SZ0112
SZ0113
SZ0115
SZ0118
SZ0119
SZ0123
SZ0124
SZ0125
SZ0127
SZ0128

SZ0129
SZ0130
SZ0131
SZ0132
SZ0133
SZ0135
SZ1044
SZ1049
SZ1050
SZ1052
SZ1053
SZ1054
SZ1055
SZ1056
SZ1057
SZ1058
SZ1059
SZ1060
SZ1061
SZ1062
SZ1063
SZ1064
SZ1065
SZ1066
SZ1067
SZ1068
SZ1069
SZ1070
SZ1071
SZ1073
SZ1074
SZ1075
SZ1076
SZ1077
SZ1078
SZ1080
SZ1081
SZ1082
SZ1083
SZ1086
SZ1087
SZ1088
SZ1090
SZ1092

SZ1093
SZ1094
SZ1095
SZ1096
SZ1098
SZ1099
SZ1100
SZ1103
SZ1104
SZ2001
SZ2003
SZ2004
SZ2005
SZ2006
SZ2007
SZ2008
SZ2009
SZ2010
SZ2012
SZ2015
SZ2022
SZ2023
SZ2024
SZ2025
SZ2027
SZ2028
SZ2029
SZ2030
SZ2031
SZ2032
SZ2033
SZ2034
SZ2035
SZ2036
SZ2037

Excluding Criteria: 3.0mm and 3.0 degree in max head motion
NC0008
NC0011
NC0018
NC0025

NC0059
NC0089
NC0092
NC0093
NC1040
NC2015
SZ0011
SZ0023
SZ0025
SZ0044
SZ0048
SZ0057
SZ0088
SZ1049
SZ1065
SZ1066
SZ1069
SZ1070
SZ1075
SZ1077
SZ1083
SZ1095
SZ1099
SZ1104
SZ2012
SZ2015
SZ2025
SZ2032
SZ2034
SZ2035

Excluding Criteria: 2.5mm and 2.5 degree in max head motion
NC0008
NC0011
NC0018
NC0025
NC0026
NC0059
NC0073
NC0089
NC0092

NC0093
NC0105
NC1036
NC1040
NC1042
NC1051
NC1078
NC2015
SZ0001
SZ0006
SZ0011
SZ0023
SZ0025
SZ0044
SZ0046
SZ0048
SZ0057
SZ0077
SZ0088
SZ0102
SZ0115
SZ0132
SZ1049
SZ1052
SZ1057
SZ1063
SZ1065
SZ1066
SZ1069
SZ1070
SZ1075
SZ1077
SZ1083
SZ1095
SZ1099
SZ1103
SZ1104
SZ2012
SZ2015
SZ2025
SZ2032
SZ2034
SZ2035

Excluding Criteria: 2.0mm and 2.0 degree in max head motion
NC0001
NC0008
NC0011
NC0018
NC0025
NC0026
NC0029
NC0033
NC0056
NC0059
NC0073
NC0077
NC0089
NC0092
NC0093
NC0095
NC0098
NC0105
NC1036
NC1040
NC1042
NC1050
NC1051
NC1054
NC1078
NC2012
NC2015
SZ0001
SZ0003
SZ0006
SZ0011
SZ0023
SZ0025
SZ0036
SZ0044
SZ0046
SZ0048
SZ0052
SZ0057
SZ0077

SZ0082
SZ0086
SZ0088
SZ0094
SZ0102
SZ0115
SZ0125
SZ0132
SZ1049
SZ1052
SZ1057
SZ1063
SZ1065
SZ1066
SZ1069
SZ1070
SZ1073
SZ1074
SZ1075
SZ1077
SZ1078
SZ1083
SZ1095
SZ1099
SZ1103
SZ1104
SZ2003
SZ2006
SZ2012
SZ2015
SZ2025
SZ2030
SZ2032
SZ2034
SZ2035

Excluding Criteria: 1.5mm and 1.5 degree in max head motion
NC0001
NC0008
NC0011
NC0013

NC0014
NC0018
NC0025
NC0026
NC0028
NC0029
NC0033
NC0035
NC0040
NC0043
NC0046
NC0052
NC0056
NC0059
NC0073
NC0077
NC0089
NC0092
NC0093
NC0094
NC0095
NC0096
NC0097
NC0098
NC0105
NC1036
NC1040
NC1041
NC1042
NC1050
NC1051
NC1054
NC1078
NC2003
NC2012
NC2015
SZ0001
SZ0003
SZ0006
SZ0007
SZ0010
SZ0011
SZ0023
SZ0025

SZ0029
SZ0036
SZ0044
SZ0046
SZ0048
SZ0052
SZ0053
SZ0057
SZ0071
SZ0077
SZ0082
SZ0086
SZ0088
SZ0094
SZ0097
SZ0102
SZ0109
SZ0113
SZ0115
SZ0118
SZ0125
SZ0132
SZ1049
SZ1052
SZ1053
SZ1055
SZ1057
SZ1063
SZ1064
SZ1065
SZ1066
SZ1067
SZ1069
SZ1070
SZ1073
SZ1074
SZ1075
SZ1077
SZ1078
SZ1083
SZ1086
SZ1094
SZ1095
SZ1099

SZ1103
SZ1104
SZ2003
SZ2006
SZ2012
SZ2015
SZ2023
SZ2025
SZ2030
SZ2031
SZ2032
SZ2034
SZ2035

Excluding Criteria: 1.0mm and 1.0 degree in max head motion
NC0001
NC0008
NC0011
NC0013
NC0014
NC0018
NC0023
NC0025
NC0026
NC0028
NC0029
NC0032
NC0033
NC0035
NC0040
NC0043
NC0046
NC0052
NC0054
NC0056
NC0057
NC0059
NC0060
NC0063
NC0068
NC0069

NC0071
NC0073
NC0074
NC0075
NC0076
NC0077
NC0088
NC0089
NC0090
NC0092
NC0093
NC0094
NC0095
NC0096
NC0097
NC0098
NC0099
NC0100
NC0105
NC0107
NC1035
NC1036
NC1038
NC1039
NC1040
NC1041
NC1042
NC1043
NC1045
NC1046
NC1049
NC1050
NC1051
NC1054
NC1055
NC1056
NC1065
NC1070
NC1078
NC1080
NC2003
NC2005
NC2010
NC2012

NC2015
NC2021
SZ0001
SZ0003
SZ0006
SZ0007
SZ0010
SZ0011
SZ0017
SZ0019
SZ0023
SZ0025
SZ0029
SZ0031
SZ0032
SZ0036
SZ0043
SZ0044
SZ0046
SZ0048
SZ0052
SZ0053
SZ0057
SZ0062
SZ0066
SZ0067
SZ0071
SZ0077
SZ0080
SZ0082
SZ0086
SZ0088
SZ0093
SZ0094
SZ0095
SZ0096
SZ0097
SZ0099
SZ0102
SZ0105
SZ0109
SZ0110
SZ0111
SZ0113

SZ0115
SZ0118
SZ0125
SZ0132
SZ1044
SZ1049
SZ1050
SZ1052
SZ1053
SZ1054
SZ1055
SZ1057
SZ1063
SZ1064
SZ1065
SZ1066
SZ1067
SZ1069
SZ1070
SZ1073
SZ1074
SZ1075
SZ1076
SZ1077
SZ1078
SZ1080
SZ1082
SZ1083
SZ1086
SZ1090
SZ1092
SZ1094
SZ1095
SZ1098
SZ1099
SZ1100
SZ1103
SZ1104
SZ2003
SZ2004
SZ2006
SZ2009
SZ2012
SZ2015

SZ2023
SZ2025
SZ2028
SZ2030
SZ2031
SZ2032
SZ2034
SZ2035

Excluding Criteria: 0.5mm and 0.5 degree in max head motion
NC0001
NC0004
NC0005
NC0007
NC0008
NC0009
NC0010
NC0011
NC0013
NC0014
NC0015
NC0016
NC0018
NC0020
NC0023
NC0025
NC0026
NC0027
NC0028
NC0029
NC0030
NC0031
NC0032
NC0033
NC0035
NC0036
NC0038
NC0039
NC0040
NC0042
NC0043

NC0044
NC0045
NC0046
NC0048
NC0050
NC0051
NC0052
NC0054
NC0056
NC0057
NC0058
NC0059
NC0060
NC0061
NC0063
NC0064
NC0065
NC0066
NC0067
NC0068
NC0069
NC0070
NC0071
NC0073
NC0074
NC0075
NC0076
NC0077
NC0078
NC0079
NC0080
NC0081
NC0083
NC0084
NC0087
NC0088
NC0089
NC0090
NC0092
NC0093
NC0094
NC0095
NC0096
NC0097

NC0098
NC0099
NC0100
NC0102
NC0103
NC0104
NC0105
NC0107
NC0108
NC1035
NC1036
NC1038
NC1039
NC1040
NC1041
NC1042
NC1043
NC1044
NC1045
NC1046
NC1048
NC1049
NC1050
NC1051
NC1052
NC1053
NC1054
NC1055
NC1056
NC1057
NC1058
NC1059
NC1060
NC1062
NC1065
NC1069
NC1070
NC1075
NC1076
NC1078
NC1079
NC1080
NC1081
NC2001

NC2003
NC2005
NC2008
NC2009
NC2010
NC2011
NC2012
NC2013
NC2015
NC2016
NC2017
NC2018
NC2019
NC2021
NC2022
NC2023
NC2024
SZ0001
SZ0003
SZ0006
SZ0007
SZ0010
SZ0011
SZ0012
SZ0015
SZ0016
SZ0017
SZ0018
SZ0019
SZ0022
SZ0023
SZ0025
SZ0027
SZ0029
SZ0031
SZ0032
SZ0036
SZ0037
SZ0038
SZ0043
SZ0044
SZ0046
SZ0048
SZ0049

SZ0050
SZ0051
SZ0052
SZ0053
SZ0054
SZ0056
SZ0057
SZ0059
SZ0061
SZ0062
SZ0064
SZ0065
SZ0066
SZ0067
SZ0069
SZ0070
SZ0071
SZ0073
SZ0076
SZ0077
SZ0079
SZ0080
SZ0082
SZ0085
SZ0086
SZ0087
SZ0088
SZ0089
SZ0091
SZ0092
SZ0093
SZ0094
SZ0095
SZ0096
SZ0097
SZ0098
SZ0099
SZ0100
SZ0102
SZ0103
SZ0105
SZ0107
SZ0109
SZ0110

SZ0111
SZ0112
SZ0113
SZ0115
SZ0118
SZ0119
SZ0123
SZ0124
SZ0125
SZ0127
SZ0128
SZ0129
SZ0130
SZ0131
SZ0132
SZ0133
SZ0135
SZ1044
SZ1049
SZ1050
SZ1052
SZ1053
SZ1054
SZ1055
SZ1056
SZ1057
SZ1058
SZ1059
SZ1062
SZ1063
SZ1064
SZ1065
SZ1066
SZ1067
SZ1068
SZ1069
SZ1070
SZ1071
SZ1073
SZ1074
SZ1075
SZ1076
SZ1077
SZ1078

SZ1080
SZ1081
SZ1082
SZ1083
SZ1086
SZ1087
SZ1088
SZ1090
SZ1092
SZ1093
SZ1094
SZ1095
SZ1096
SZ1098
SZ1099
SZ1100
SZ1103
SZ1104
SZ2001
SZ2003
SZ2004
SZ2005
SZ2006
SZ2007
SZ2008
SZ2009
SZ2010
SZ2012
SZ2015
SZ2022
SZ2023
SZ2024
SZ2025
SZ2027
SZ2028
SZ2030
SZ2031
SZ2032
SZ2033
SZ2034
SZ2035
SZ2037
