# Supplementary material for: Reduced inter-hemispheric auditory and memory-related network interactions in patients with schizophrenia experiencing auditory verbal hallucinations
Source: Front Psychiatry. 2022 Aug 3;13:956895. doi: 10.3389/fpsyt.2022.956895 (PMC9381966; doi:10.3389/fpsyt.2022.956895)
Supplement: Supplementary file 2 [file Data_Sheet_2.PDF]

| Subject ID | mean FD_Jenkinson |
|---|---|
| NC0002 | 0.02732236 |
| NC0003 | 0.04878803 |
| NC0004 | 0.04051368 |
| NC0005 | 0.03934293 |
| NC0006 | 0.03735516 |
| NC0007 | 0.04097846 |
| NC00010 | 0.09308458 |
| NC00012 | 0.04756252 |
| NC00013 | 0.2064846 |
| NC00014 | 0.0692375 |
| NC00016 | 0.04385411 |
| NC00017 | 0.03324171 |
| NC00019 | 0.02472299 |
| NC00021 | 0.05440484 |
| NC00022 | 0.02727109 |
| NC00023 | 0.05430875 |
| NC00026 | 0.04423548 |
| NC00027 | 0.01734374 |
| NC00028 | 0.06943873 |
| NC00029 | 0.08121666 |
| NC00030 | 0.05195999 |
| NC00031 | 0.06280608 |
| NC00032 | 0.03080883 |
| NC00033 | 0.06294053 |
| NC00034 | 0.07372041 |
| NC00035 | 0.07216206 |
| NC00036 | 0.04416822 |
| NC00037 | 0.05907444 |
| NC00038 | 0.04881532 |
| NC00039 | 0.07176399 |
| NC00040 | 0.04201835 |
| NC00041 | 0.1632832 |
| NC00042 | 0.03129692 |
| NC00043 | 0.1082787 |
| NC00044 | 0.09584973 |
| NC00045 | 0.03105022 |
| NC00047 | 0.0629348 |
| NC00048 | 0.05626022 |
| NC00049 | 0.05303214 |
| NC00050 | 0.04449836 |
| NC00052 | 0.06143286 |
| NC00053 | 0.03776392 |

| | |
|---|---|
| NC00054 | 0.07919077 |
| NC00055 | 0.03704155 |
| NC00056 | 0.08850359 |
| NC00057 | 0.05836395 |
| NC00058 | 0.03595435 |
| NC00060 | 0.05384734 |
| NC00062 | 0.04164923 |
| NC00063 | 0.1222586 |
| NC00064 | 0.02714362 |
| NC00065 | 0.02276456 |
| NC00067 | 0.03987392 |
| NC00068 | 0.05059125 |
| NC00069 | 0.03011811 |
| NC00070 | 0.02649294 |
| NC00071 | 0.03155675 |
| NC00073 | 0.08889745 |
| NC00074 | 0.04941199 |
| NC00075 | 0.06621533 |
| NC00076 | 0.04569955 |
| NC00078 | 0.1112548 |
| NC00079 | 0.05114126 |
| NC00080 | 0.04000454 |
| NC00081 | 0.03560124 |
| NC00082 | 0.03104917 |
| NC00083 | 0.03588439 |
| NC00084 | 0.03099306 |
| NC00085 | 0.02494262 |
| NC00086 | 0.0545959 |
| NC00087 | 0.09074007 |
| NC00088 | 0.07424945 |
| NC00090 | 0.06526214 |
| NC00091 | 0.0381358 |
| NC00094 | 0.03901343 |
| NC00095 | 0.08198688 |
| NC00096 | 0.07451156 |
| NC00097 | 0.08024824 |
| NC00098 | 0.1306528 |
| NC00099 | 0.06783894 |
| NC00100 | 0.02376572 |
| NC00103 | 0.03429947 |
| SZ0004 | 0.0238124 |
| SZ0010 | 0.1207758 |
| SZ0015 | 0.03358835 |

| | |
|---|---|
| SZ0017 | 0.03468338 |
| SZ0018 | 0.04055373 |
| SZ0022 | 0.08179229 |
| SZ0031 | 0.1017966 |
| SZ0038 | 0.07014031 |
| SZ0039 | 0.02524754 |
| SZ0043 | 0.04757257 |
| SZ0046 | 0.4232924 |
| SZ0053 | 0.07507023 |
| SZ0056 | 0.02877257 |
| SZ0060 | 0.1506465 |
| SZ0062 | 0.08725193 |
| SZ0063 | 0.0276201 |
| SZ0064 | 0.05107075 |
| SZ0065 | 0.03029877 |
| SZ0066 | 0.1475319 |
| SZ0068 | 0.02316146 |
| SZ0070 | 0.04576936 |
| SZ0073 | 0.06923854 |
| SZ0074 | 0.02360309 |
| SZ0075 | 0.03597058 |
| SZ0076 | 0.05490648 |
| SZ0077 | 0.1081363 |
| SZ0078 | 0.02313065 |
| SZ0079 | 0.04482244 |
| SZ0080 | 0.02568325 |
| SZ0083 | 0.1842492 |
| SZ0085 | 0.09666549 |
| SZ0091 | 0.03627859 |
| SZ0094 | 0.1738868 |
| SZ0095 | 0.07835668 |
| SZ0099 | 0.04536451 |
| SZ0104 | 0.04940195 |
| SZ0107 | 0.03628528 |
| SZ0111 | 0.07102981 |
| SZ0112 | 0.09606415 |
| SZ0113 | 0.0512571 |
| SZ0115 | 0.05652316 |
| SZ0126 | 0.02432783 |
| SZ0003 | 0.0744847 |
| SZ0027 | 0.05842891 |
| SZ0029 | 0.1199825 |
| SZ0030 | 0.02025187 |

| | |
|---|---|
| SZ0058 | 0.02670222 |
| SZ0067 | 0.03068087 |
| SZ0071 | 0.05160786 |
| SZ0072 | 0.02406783 |
| SZ0080 | 0.03314263 |
| SZ0082 | 0.161353 |
| SZ0087 | 0.0214649 |
| SZ0092 | 0.07430766 |
| SZ0093 | 0.07369463 |
| SZ0096 | 0.08686142 |
| SZ0097 | 0.2060332 |
| SZ0102 | 0.1682719 |
| SZ0105 | 0.04698066 |
| SZ0109 | 0.04909247 |
| SZ0110 | 0.04926798 |
| SZ0116 | 0.02905195 |
| SZ0118 | 0.07534813 |
| SZ0119 | 0.07114733 |
| SZ0120 | 0.04526319 |
| SZ0123 | 0.1102791 |
| SZ0125 | 0.04277574 |
| SZ0133 | 0.07920335 |
